# Targeted Therapy of Advanced Gallbladder Cancer and Cholangiocarcinoma with Aggressive Biology: Eliciting Early Response Signals from Phase 1 trials

**DOI:** 10.18632/oncotarget.832

**Published:** 2013-01-27

**Authors:** Ishwaria M. Subbiah, Vivek Subbiah, Apostolia M. Tsimberidou, Aung Naing, Ahmed O. Kaseb, Milind Javle, Siqing Fu, David S. Hong, Sarina Piha-Paul, Jennifer J. Wheler, Kenneth R. Hess, Filip Janku, Gerald S. Falchook, Robert A Wolff, Razelle Kurzrock

**Affiliations:** ^1^ Department of Investigational Cancer Therapeutics; ^2^ Department of Gastrointestinal Medical Oncology, Division of Cancer Medicine; ^3^ Department of Biostatistics, The University of Texas MD Anderson Cancer Center, Houston, TX; ^4^ Moores Cancer Center, University of California, San Diego, California

**Keywords:** gallbladder carcinoma, cholangiocarcinoma, phase I, targeted therapy, locoregional therapy

## Abstract

**Purpose:**

Patients with advanced cholangiocarcinoma (CC) and gallbladder carcinoma (GC) have few therapeutic options for relapsed disease.

**Methods:**

Given the overall poor prognosis in this population and the availability of novel targeted therapies, we systematically analyzed the characteristics and outcomes for GC and CC patients treated on phase I trials with an emphasis on targeted agents and locoregional therapies.

**Results:**

Of 40 treated patients (GC=6; CC=34; median age, 60 years), 8 (20%) had stable disease (SD) &gt; 6 months, 3 (8%) partial response (PR), on protocols with hepatic arterial drug infusion and anti-angiogenic, anti-HER-2/neu or novel MAPK/ERK kinase (MEK) inhibitors. Median progression-free survival (PFS) on phase I trials was 2.0 months (95% CI 1.7, 2.8) versus 3.0 months (95% CI 2.4, 5.0), 3.0 months (95% CI 2.3, 4.6), and 3.0 months (95% CI 2.4, 3.9) for their first-, second-, and last-line FDA-approved therapy. In univariate analysis, &gt;3 metastatic sites, elevated alanine aminotransferase (ALT) (&gt;56IU/L), serum creatinine (&gt;1.6mg/dL), and CA19-9 (&gt;35U/mL) were associated with a shorter PFS. Mutational analysis revealed mutation in the KRAS oncogene in 2 of 11 patients (18%). The SD &gt;6 months/PR rate of 28% was seen with hepatic arterial infusion of oxaliplatin, and inhibitors of angiogenesis, HER-2/neu or MEK.

**Conclusions:**

The PFS in phase I trials was similar to that of the first, second, and last-line therapy (P=0.95, 0.98, 0.76, respectively) with FDA-approved agents given in the advanced setting, emphasizing a role for targeted agents in a clinical trials setting as potentially valuable therapeutic options for these patients.

## INTRODUCTION

Cholangiocarcinoma (CC) and gallbladder carcinoma (GC) are rare, but aggressive, biliary tract malignancies characterized by their advanced stage at diagnosis and high mortality. Though anatomically neighbors, GC and CC are histologically distinct entities arising from the epithelium of the gallbladder and hepatic bile ducts, respectively.[1] CC is further stratified by anatomic location, either intrahepatic or extrahepatic. CC and GC are challenging diseases to diagnose given their insidious evolution and lack of effective screening modalities. With few cases (<10%) presenting as early disease, most patients are initially diagnosed with metastatic disease that is not amenable to surgery.[2] Despite advancements in the multimodal approach to the treatment of advanced solid tumors, the prognosis of advanced CC and GC remains grim.[2-4] Unfortunately, epidemiological trends point towards an increasing incidence and prevalence of CC and GC in East Asia, Latin America and in western societies, particularly given their association with hepatitis C infection, chronic non-alcoholic fatty liver disease and obesity.[5, 6] Current Food and Drug Administration (FDA) approved agents used in unresectable CC and GC include gemcitabine, capecitabine, cisplatin, oxaliplatin, fluoropyrimidines, including 5-fluorouracil, or a combination of these agents, although none are FDA approved primarily for use in biliary tract cancers. [7, 8] Recent protocols combine chemotherapy with radiation either in the preoperative neoadjuvant setting or in the postoperative setting. Even multi-agent chemotherapeutic approaches do not confer a durable benefit in patients with metastatic and/or relapsed disease, and fewer than 5% of patients survive 5 years after advanced diagnosis.[9]

We embarked on this study to analyze the characteristics and outcomes of patients with advanced CC and GC with very aggressive biology referred for phase I clinical trials at a major cancer center with an emphasis on targeted agents to elicit early response signals.

## PATIENTS AND METHODS

We reviewed the characteristics and outcomes of 72 consecutive patients with GC and CC referred to the Clinical Center for Targeted Therapy (Phase I Clinical Trials Program) at MD Anderson Cancer Center starting from November 2004. Eligibility criteria for participation in phase I clinical trials included age >18 years, presence of metastatic or unresectable disease, measurable disease per Response Evaluation Criteria in Solid Tumors (RECIST) 1.0,[10] Eastern Cooperative Oncology Group (ECOG) performance status (PS) of 0-1,[11] and a life expectancy >3 months. Premenopausal women were required to have a negative pregnancy test and patients of childbearing potential to use contraception. Further eligibility criteria varied according to the particular study and all patients gave informed consent. This study and all clinical trials were approved by the MD Anderson Institutional Review Board.

After review of baseline clinical, laboratory, radiologic and pathologic data during the initial consultation, patients were enrolled on a phase I trial based on scientific rationale and protocol availability. After initiation of an investigational therapy, patients were evaluated at 2- to 4- week intervals, based on the specific protocol, with a medical history, physical examination, comprehensive series of laboratory tests, and assessments of toxicity and compliance. Restaging scans were done every 6-8 weeks, depending on the protocol. Obstructive cholestasis were managed according to the patients performance status and clinical status by interventional radiologist intervention or a stent placement by the gastro-intestinal consult team.

### Endpoints and Statistical Methods

All statistical analysis was performed by our biostatistician (K.R.H.). Descriptive statistics summarized the patients’ characteristics. Cox proportional hazards regression analysis was used to examine the association between progression-free survival (PFS) since beginning phase I therapy and the following variables measured at the time of initial phase I consultation: age at diagnosis, gender, ECOG PS, number of metastatic sites, number of prior therapies, hemoglobin, lactate dehydrogenase (LDH), platelet count, history of thromboembolism, total bilirubin, aspartate aminotransferase (AST), alanine aminotransferase (ALT), alkaline phosphatase, serum sodium, serum creatinine, tumor markers (carcinoembryonic antigen [CEA], carbohydrate antigens [CAs] CA 19-9, CA 125, CA 27.29), presence of ascites, serum albumin, and the Royal Marsden Hospital (RMH) prognostic score (comprised of serum albumin <3.5 g/dL, lactate dehydrogenase > upper limit of normal and > 2 metastatic sites).[12]

Best response was assessed using RECIST 1.0 every 2 cycles (6-8 weeks) of therapy as per the protocol.[10] Partial response (PR) was defined as a >30% decrease in the sum of the longest diameter of target lesions, excluding complete disappearance of disease (complete response, CR) and progressive disease (PD) was a > 20% increase. Stable disease (SD) was defined as changes that did not meet the criteria for a PR or PD. Waterfall plot analysis according to RECIST is used to illustrate response.

Overall survival was measured from the date of enrollment on a phase I trial until death from any cause or date of last follow-up. PFS is defined as the time from first day of treatment on a phase I trial to date when treatment on the last phase I trial ceased due to disease progression or death, but not toxicity. Patients still alive (in case of survival analysis) and free of progression (in case of PFS analysis) were censored at time of last follow-up. Toxicities were assessed using the National Cancer Institute Common Terminology Criteria for Adverse Events, version per the protocol.[13] A *P*-value <.05 was considered statistically significant. Statistical analysis was performed using S-PLUS® 8.0 for Windows (Insightful Corp.).

## RESULTS

### Patients Characteristics

Of the 72 patients with GC and CC referred to the Phase I Clinical Trials program, 32 patients (30 CC and 2 GC) were not enrolled in a phase I clinical trial due to deterioration of performance status (*N*=25), decision to pursue alternate therapies including treatments closer to home (*N*=6), and insurance denial (*N*=1). Forty patients who participated in a phase I trial are included in this analysis. Pretreatment characteristics at presentation to the Phase I Clinic are summarized in Table 1. The median age at diagnosis was 60 years (range, 41.4-73.6 years). There were 23 women and 17 men. Thirty-one (78%) were White, 5 (12%) were African American, and 4 (10%) were Hispanic. Seven patients (18%) had an ECOG PS of 0, 29 patients (72%) had a PS of 1 and 4 (10%) patients a 2. Of the 40 patients treated, 6 had gallbladder carcinoma, 4 had extrahepatic cholangiocarcinoma and 30 had intrahepatic cholangiocarcinoma; the tumor histology for all 40 patients was consistent with adenocarcinoma. The median number of metastatic sites was 3 (range 0-6). The most common sites of metastases at time of phase I referral were liver (90% of patients), lymph nodes (83%), peritoneum (40%), retroperitoneum (38%) and lung (38%). The median time from initial phase I consultation to day one on a phase I trial was 16 days.

**Table 1:** Patient Characteristics

		N	%
Sex			
	Male	17	42.5%
	Female	23	57.5%
Age at time of diagnosis,			
	Median (range)	60.2	(21.6 - 73.6)
	Age >60	21	52.5%
	Age <60	19	47.5%
Race			
	White	31	77.5%
	African American	5	12.5%
	Hispanic	4	10.0%
ECOG Performance Status			
	0-1	36	90.0%
	2-3	4	10.0%
Tumor histology			
	Cholangiocarcinoma	34	85.0%
	Intrahepatic	30	
	Extrahepatic	4	
	Gallbladder carcinoma	6	15.0%
Number of metastatic sites			
	Median (range)	3	0 - 6
	0-2	10	25.0%
	> 3	30	75.0%
Metastatic sites			
	Liver	36	90.0%
	Lymph nodes	33	82.5%
	Peritoneum	15	40.0%
	Lung	15	37.5%
	Retroperitoneum	15	37.5%
	Bone	6	15.0%
	Pancreas	5	12.8%
	Spleen	4	10.0%
	Kidney	1	2.5%
	Ovary	1	2.5%
	Adrenal	1	2.5%
Prior treatments			
	Prior surgical resection	15	37.5%
	Prior chemoembolization	3	7.5%
	Prior radiation	13	32.5%
Number of prior systemic therapies			
	Median (range)	3	(0-11)
	0-5	31	77.5%
	>5	9	22.5%

### Therapy before patient inclusion in phase I trials

Overall, of the 40 patients enrolled on a phase I trial, two patients had received no prior therapy because of the unavailability of reasonable, conventional therapy for the extent of their disease. The remaining 38 patients had a median of 3 prior systemic therapies before referral to the Phase I Clinic (range, 1 – 11). Fifteen patients (38%) also underwent a prior surgical resection, whereas 13 (33%) received prior radiation and 3 (8%) had prior chemoembolization.

The National Comprehensive Cancer Network (NCCN) guidelines for unresectable gallbladder and cholangiocarcinoma include a gemcitabine/cisplatin combination therapy, fluoropyrimidine-based or other-gemcitabine based regimen.[4, 14] For their first-line treatment in the advanced/metastatic setting, 4 of 38 patients received experimental therapy on phase II trials with a novel paclitaxel conjugate or a campotothecin analog. The remaining 34 patients received a first-line regimen based on gemcitabine (*N*=24, 71%), a fluoropyrimidine (*N*=7, 21%), or single-agent sorafenib (*N*=3, 8%). As their second-line therapy in the advanced disease setting, 36 patients received a regimen including gemcitabine (*N*=17, 47%), a fluropyrimidine (*N*=16, 45%), or a targeted agent (*N*=3, 8%); 2 patients did not received a second line treatment and instead began phase I therapy. All 38 patients were treated on a regimen based on FDA-approved drugs as their last antineoplastic therapy before beginning a phase I trial; 58% received a fluoropyrimidine-based regimen (26% received fluoropyrimidine agents in combination with platinum), whereas 23% received a targeted agent, most commonly an angiogenesis inhibitor. Thus FDA-approved agents formed the backbone of the systemic combination therapy prior to enrollment in a phase I trial.

### Treatment

Overall, patients were initially treated on 1 of 22 phase I clinical trials; of these trials, 8 patients received therapy on six first-in-human trials with novel targeted inhibitors against MEK, VEGF, gamma-secretase, aurora kinase and the IGF-IR pathway. Of 40 patients, 30 (75%) were treated on a trial with combination therapy of two or more agents while 10 patients (25%) received treatment with a single agent. Seventeen (43%) patients received locoregional treatment with direct infusion of a cytotoxic agent into the hepatic artery; 24 (60%) received intravenous angiogenesis inhibitors, of whom 13 (33%) received the antiangiogenic agent in combination with hepatic arterial infusion therapy. Fifteen (38%) patients received targeted agent(s) alone, 20 (50%) received targeted agent(s) in combination with cytotoxic chemotherapy, and 5 (12%) received cytotoxic chemotherapy alone. The median number of cycles received was 2 (range 1 – 12). Six patients went on to receive therapy under a second phase I trial; of these patients, 3 received treatment on 3 phase I trials.

### Response

**Figure 1:**
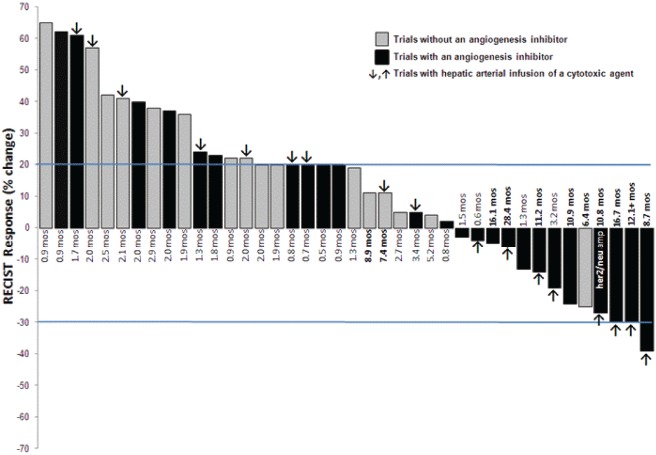
Waterfall plot of the best RECIST response to the best phase I trial of all 40 treated patients Six patients who did not undergo restaging due to early disease progression are reflected as a 20% increase. Time (in months) reflects the duration of response.

**Figure 2:**
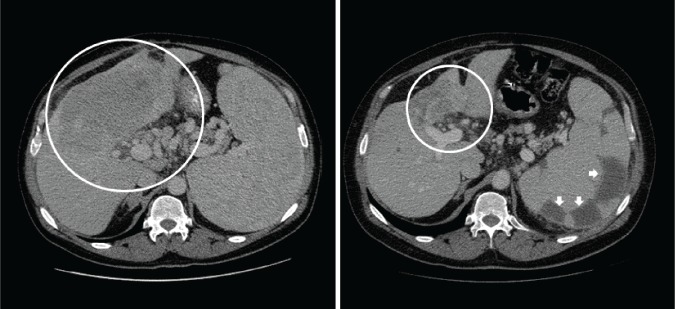
Prolonged partial response for 12.1 months noted in the primary hepatic lesion of a patient with advanced cholangiocarcinoma treated with hepatic arterial infusion of a cytotoxic agent along with intravenous anti-angiogenic agent CT scan of the abdomen showed liver metastases at baseline and after 12 months of treatment. White arrows show multiple areas of hypoattenuation secondary to splenic embolization.

Six patients were not restaged prior to end of cycle 2 due to clinical deterioration and early disease progression. Of the 40 patients treated on clinical studies, 3 (8%) had a partial response (PR), 17 had stable disease (43%) including 8 (20%) who had SD > 6 months, and 20 patients had progressive disease (PD) with a SD> 6 months/PR rate of 27.5% (Figure 1). The highest rates of prolonged SD > 6 months/PR were observed in patients treated with protocols that included hepatic arterial infusion (HAI) of oxaliplatin or paclitaxel (7 of 17 patients treated on HAI regimens, 41%) or antiangiogenic agents (9 of 24 patients, 38%) with 6 of those patients receiving treatment that included HAI of oxaliplatin combined with systemic bevacizumab (Figure 2; Table 3). A prolonged SD of 10.8 months was observed in a patient with metastatic GC with HER-2/neu amplification who was treated on phase I protocols, first combining the anti-HER-2/neu antibody trastuzumab and the HER-2/neu tyrosine kinase inhibitor lapatinib, and then with trastuzumab and erlotinib. Prolonged SD lasting 9 months was also seen in one patient treated on a first-in-human trial using a novel MEK inhibitor.

### Survival and toxicities

**Figure 3:**
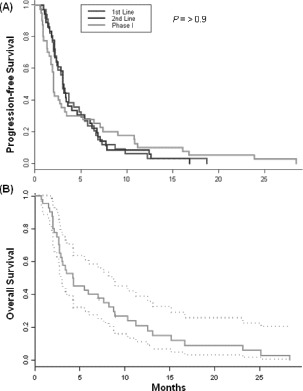
(A) PFS of patients treated on phase I trials compared to their first-line, second-line and last systemic antitumor therapy given in the advanced setting prior to phase I referral. (B) Median overall survival after starting a phase I trial. Dotted lines represent 95% confidence intervals for the estimated survival probabilities.

For the 40 patients treated on phase I trials, the time from diagnosis of advanced (metastatic) disease to primary evaluation in the Phase I Clinical Center for Targeted Therapy was 13.1 months. The median time from the initial phase I consultation to day 1 of a trial was 16 days. The overall median survival from day 1 on a phase I trial was 4.2 months (95% CI 3.8 – 8.6 months). Median PFS for 40 treated patients was 2.0 months (95% CI 1.8, 3.4) on phase I clinical trials. Among these 40 patients, the median PFS on their first-line and second-line prior therapies with FDA-approved agents given in the advanced setting was 3.0 months (95% CI 2.4, 5.0) and 3.0 months (95% CI 2.3, 4.6), respectively. Median PFS on their last FDA-approved treatment before phase I referral was 3.0 months (95% CI 2.4, 3.9). In comparison, the median PFS on phase I therapy did not differ significantly from that on their first-line or second-line FDA-approved agents given for advanced disease or their last treatment prior to phase I referral (*P*=0.95, 0.98, 0.76, respectively) (Figure 3A). Data were also analyzed from the 32 patients who were not enrolled on phase I trials and the PFS of their first-, second-line, and last treatment with FDA-approved agents prior to phase I referral (3.1, 2.4, 3.0 months, respectively) also did not differ significantly from the PFS of the phase I-treated patients.

Overall survival from enrollment on a phase I trial to date of death is shown in Figure 3B. Among the 40 treated patients, 38 (95%) had died at the time of analysis. The 90-day mortality was 38% with 25 patients alive at 3 months after beginning phase I therapy; the 6-month mortality was 63% with 15 patients alive at 6 months after beginning therapy on phase I trials. Importantly, there was no treatment-related mortality. Two patients treated on different combination regimens that included an angiogenesis inhibitor experienced grade 3 gastrointestinal bleeding, prompting a change of regimen, with overall progression-free period of 10.8 and 16.8 months, respectively, on their subsequent phase I trials. The only other grade 3 toxicity that prompted removal from study was the development of an arteriovenous fistula in one patient due to the placement of the hepatic arterial infusion catheter.

### Prognostic Factors for Survival

We conducted univariate analysis to evaluate the effects on survival of variables including age, sex, race/ethnicity, ECOG performance status, tumor markers (carcinoembryonic antigen [CEA], carbohydrate antigens [CAs] CA 19-9, CA 125, CA 27.29); history of thromboembolism; number of prior therapies; presence of liver metastases; number of metastatic sites; leukocyte count; hemoglobin level; platelet count; and albumin, lactate dehydrogenase (LDH), alkaline phosphatase, bilirubin, alanine aminotransferase, aspartate aminotransferase, RMH score and serum creatinine levels (Table 2). In univariate analysis, factors associated with a shorter Phase I PFS were a higher number of metastatic sites (1-2 versus >3; hazard ratio [HR] 3.2, *P*=0.070), elevated serum ALT (> 56 IU/L; HR 5.3, *P*=0.007), elevated serum creatinine (>1.6 mg/dL; *P*=0.022), and high CA19-9 (>35 U/mL; HR 4.8, *P*=0.010).

**Table 2:** Univariate Proportional Hazards Regression Model for Phase I Progression Free Survival (PFS)

	N	Median PFS (mos)	Hazard ratio	P-value
Gender			0.5 (0.3, 2.1)	
Male : Female	17 : 23	2.9 : 2.5		0.75
Age (yrs)			0.7 (0.3, 1.8)	0.5
< 60 : >60	19 : 21	2.5 : 2.1		
ECOG PS			2.2 (0.6, 7.5)	0.26
PS 0-1 : 2	36 : 4	2.5 : 1.4		
Number of metastatic site			3.2 (0.7, 1.4)	0.07
1-2 : > 3	10 : 30	NR : 2.0		
Number of prior therapies			0.9 (0.3, 2.5)	0.82
< 5 : > 5	31 : 9	2.1 : 2.9		
Hemoglobin (g/dL)			1.7 (0.7, 4.0)	0.26
< 10.5 : > 10.5	25 : 15	NR : 2.0		
LDH (IU/L)			1.0 (0.4, 2.5)	0.99
< 618 : > 618	27 : 13	2.5 : 2.0		
Platelet count (K/UL)			n/a	n/a
< 439 : > 439	39 : 1	2.5 : NR		
History of thromboembolism			1.1 (0.4, 3.0)	0.86
Yes : No	11 : 29	2.5 : 2.0		
Total bilirubin (mg/dL)			1.6 (0.6, 4.2)	0.33
< 1: > 1	30 : 10	2.9 : 2.0		
ALT (IU/L)			5.3 (1.8, 16)	0.0072
< 56 : > 56	35 : 5	NR : 1.3		
AST (IU/L)			1.0 (0.4, 2.3)	0.91
< 46 : > 46	21 : 19	2.1 : 2.9		
Alkaline phosphatase (IU/L)			0.6 (0.2, 1.5)	0.3
< 126 : > 126	12 : 28	2.0 : 2.9		
Serum sodium (mEq/L)			1.4 (0.4, 4.6)	0.64
> 135 : < 135	36 : 4	2.1 : 2.4		
Serum creatinine (mg/dL)			n/a	0.022
< 1.6 : > 1.6	36 : 4	2 : NR		
CEA (ng/mL)			1.2 (0.4, 3.8)	0.69
< 6 : > 6	33 : 7	2.5 : 1.8		
CA-19-9 (U/ml)			4.8 (1.1, 21)	0.01
< 35 : > 35	11 : 29	NR : 2.0		
CA 125 (U/ml)			1.1 (0.4, 2.6)	0.87
< 35 : > 35	20 : 20	2.0 : 2.9		
CA 27.29 (U/ml)			1.2 (0.5, 3.0)	0.66
< 47 : > 47	22 : 16	2.5 : 2.0		
Presence of ascites			1.1 (0.5, 2.7)	0.79
Yes : No	19 : 21	2.1 : 2.9		
Serum albumin (g/dL)			1.5 (0.6, 3.8)	0.44
< 3.5 : > 3.5	29 : 11	2.5 : 2.0		
RMH score			1.5 (0.6, 3.7)	0.34
0-1 : 2-3	21 : 19	2.9 : 2.0		

Abbreviations: ALT alanine aminotransferase; AST aspartate aminotransferase; ECOG, Eastern Cooperative Oncology Group; LDH lactate dehydrogenase; CEA Carcinoembryonic antigen; CA carbohydrate antigen; NR not reached; PS, performance status; RMH Royal Marsden Hospital; n/a Not applicable

**Table 3:** Characteristics of patients with clinical benefit rate (PR + SD > 6months)

Pt	Age/Gender	Diagnosis	Phase I Regimen(s)	Best response	PFS on Phase I trial (months)
1	57/F	Intrahepatic CC	HAI Oxaliplatin + IV Bevacizumab + IV 5FU	PR on Phase I, NED post resection**	16.7
22	55/M	Intrahepatic CC	HAI Oxaliplatin + IV 5FU + IV Bevacizumab + IV Cetuximab	PR	9+
34	47/F	Intrahepatic CC	HAI Nab-Paclitaxel + IV Gemcitabine + IV Bevacizumab	PR	7.1+
8	55/F	Intrahepatic CC	HAI Oxaliplatin + IV Bevacizumab + IV 5FU	SD	6.4
37	69/F	Intrahepatic CC	HAI Oxaliplatin + IV Bevacizumab + IV 5FU	SD	7.2
26	71/F	Extrahepatic CC	HAI Nab-Paclitaxel	SD	7.4
36	61/F	Gallbladder carcinoma	Bevacizumab + Trastuzumab + Lapatinib; Trastuzumab + Erlotinib	SD	10.4
25	47/M	Intrahepatic CC	Bevacizumab + Sorafenib	SD	10.9
39	67/M	Intrahepatic CC	HAI Oxaliplatin + IV Bevacizumab + IV 5FU	SD	11.3
38	60/F	Intrahepatic CC	Bevacizumab + Sorafenib; Sirolimus + Cetuximab	SD	14.3
15	53/M	Intrahepatic CC	Novel MEK inhibitor	SD	8.9

5-FU fluorouracil; CC cholangiocarcinoma; CR complete response; HAI hepatic arterial infusion; PR partial response; SD stable disease

** This patient went on to receive neoadjuvant bevacizumab + proton therapy, followed by an extended hepatectomy, ultimately showing no evidence of disease (NED).

**Table 4:** Molecular analyses of the 40 treated patients treated on Phase I trials

Molecular mutation or aberration	Number of patients tested	Number of patients with a mutation or aberration
KRAS	11	2
NRAS	7	0
BRAF	10	0
CKIT	3	0
EGFR	10	0
PI3K	9	0
GNAQ	1	0
PTEN Loss	3	0
ER IHC	6	0
PR IHC	6	0
HER-2/neu FISH	8	1

Abbreviations: IHC immunohistochemistry; FISH fluorescence in situ hybridization

### Molecular analysis

Testing for mutations in *KRAS, NRAS, BRAF, cKIT, EGFR, PIK3CA, TP53*, as well as immunohistochemistry for PTEN loss and HER-2/neu FISH amplification was completed on patients with adequate available tissue in the MD Anderson CLIA-certified laboratory. DNA was extracted from micro-dissected paraffin-embedded tumor and analyzed by a PCR-based DNA sequencing method to examine codons 12, 13 and 61 of the *KRAS* proto-oncogene. Of 11 tested patients, a *KRAS* mutation was detected in the tumor of two patients with cholangiocarcinoma, one in codon 12 and another in codon 13.[15] A third patient's gallbladder carcinoma demonstrated FISH amplification of HER-2/neu (HER-2/neu: CEP17 signal ratio: 6.49). This patient maintained SD for 27 months with a HER-2/neu targeting agent prior to progression; then, phase I therapy including HER-2/neu targeting agents resulted in SD for an additional 10.8 months.

## DISCUSSION

Treatment options for patients with advanced unresectable GC and CC are limited given the poor outcomes of even frontline therapies with FDA-approved agents. Complete surgical resection remains the only curative modality of therapy, offering benefit only for patients with localized disease. The NCCN guidelines for relapsed GC and CC highlight the limited clinical trial data that can be used to define a standard regimen or definitive benefit with an emphasis on clinical trial participation.[3, 16]

Approximately 44% of patients with CC and GC referred for phase I trials were ineligible, mainly because of poor performance status (ECOG PS 3 or greater). Our analysis shows 28% of our patients achieved SD > 6months/PR mainly with locoregional therapy with hepatic arterial infusion and angiogenesis inhibitors, suggesting that these modalities merit further investigation in similar patients. Additionally, the fact that SD > 6months was seen with targeted therapies including a MEK inhibitor (SD 9 months) and an HER-2/neu targeting agent (SD 11 months) may be of investigational interest. Indeed, Bendell and colleagues also demonstrated a PR in cholangiocarcinoma treated with a MEK 1/2 inhibitor.[17] Although *KRAS* mutations may sensitize a tumor to MEK inhibitors,[18] the *KRAS* status of our patient with prolonged stable disease on a novel MEK inhibitor was unknown due to lack of tissue available for molecular analysis.

Overall, the pattern of molecular aberrations seen in GC and CC is slowly emerging. Somatic mutations of *KRAS*, most commonly G12D and G13D, and *PIK3CA*, most commonly E545K, along with deletion of *p53* have been reported in CCs; activating mutations in *PIK3CA* have also been reported in 12.5% of patients with GC, highlighting the therapeutic targeting potential of the RAS/RAF/MEK/ERK pathway and PI3K/AKT/MTOR pathway.[19, 20] It is conceivable that the responses to MEK inhibitors seen in CC are due to the frequency of *KRAS* mutations among these patients (13%).[20] Most recently, tissue from 9 of 40 patients (23%) with intrahepatic CC was shown to harbor mutations in the gene encoding isocitrate dehydrogenase 1 (IDH1).[21] The clinical significance of this is not known, but one proposed mechanism demonstrates that a heterozygous *IDH1* mutation leads to elevated levels of hypoxia-inducible factor subunit HIF-1alpha, thereby promoting tumor development and suggesting that IDH1 may be a tumor suppressor. We have recently shown that better response rates, time to treatment failure, and overall survival are associated with matching therapy to actionable mutations, highlighting the importance of further assessment of this strategy in these populations.[22]

Our analysis demonstrates that the PFS of phase I therapy in our program is comparable to that of the first-line, second-line and last prior treatment options with FDA-approved agents for advanced disease. There are several limitations to our analysis. First, a selection bias exists within our patient population given that the patients presented with widely metastatic disease and the PFS on frontline therapies in this population, particularly following treatment with the gemcitabine/cisplatin combination, was shorter than that reported in the pivotal phase III trial.[14] Such findings may suggest a more aggressive tumor behavior that has yet to be characterized among our patients. Second, a substantial number of patients (32 of 72 referred patients, 44%) were not enrolled on a phase I trial. This was due, for the most part, to clinical deterioration, although the first-, second-, and last line PFS among these untreated patients did not differ from that of the 40 patients treated on a phase I trial. Thirdly, we were unable to conduct an effective multivariate analysis given the small sample size. Finally, mutational analysis was only performed in a small subset of patients due to the lack of tissue availability for testing. Additionally, data on quality of life as an endpoint was not collected and, given the poor prognosis of these patients, an emphasis on quality of life measurements becomes critical. Future clinical trials should include quality of life as an important end point.

Predictors of shorter phase I PFS in univariate analysis were a higher number of metastatic site (>3), elevated serum ALT (>56 IU/L), elevated serum creatinine (>1.6 mg/dL), and high CA19-9 (>35 U/mL). Overall, the short PFS reflects the rather dismal outcomes of these advanced GC and CC patients and emphasizes the need for new approaches. Since 28% of our patients who were able to enroll of a trial achieved SD > 6 months/PR mainly on our trials with locoregional therapy, anti-angiogenic agents, MEK and HER-2/neu inhibitors, these strategies may merit further exploration.
